# Interaction of Species Traits and Environmental Disturbance Predicts Invasion Success of Aquatic Microorganisms

**DOI:** 10.1371/journal.pone.0045400

**Published:** 2012-09-20

**Authors:** Elvira Mächler, Florian Altermatt

**Affiliations:** 1 Department of Aquatic Ecology, Eawag: Swiss Federal Institute of Aquatic Science and Technology, Dübendorf, Switzerland; 2 Department of Biology, ETH Zurich, CHN, Zürich, Switzerland; University of Zurich, Switzerland

## Abstract

Factors such as increased mobility of humans, global trade and climate change are affecting the range of many species, and cause large-scale translocations of species beyond their native range. Many introduced species have a strong negative influence on the new local environment and lead to high economic costs. There is a strong interest to understand why some species are successful in invading new environments and others not. Most of our understanding and generalizations thereof, however, are based on studies of plants and animals, and little is known on invasion processes of microorganisms. We conducted a microcosm experiment to understand factors promoting the success of biological invasions of aquatic microorganisms. In a controlled lab experiment, protist and rotifer species originally isolated in North America invaded into a natural, field-collected community of microorganisms of European origin. To identify the importance of environmental disturbances on invasion success, we either repeatedly disturbed the local patches, or kept them as undisturbed controls. We measured both short-term establishment and long-term invasion success, and correlated it with species-specific life-history traits. We found that environmental disturbances significantly affected invasion success. Depending on the invading species’ identity, disturbances were either promoting or decreasing invasion success. The interaction between habitat disturbance and species identity was especially pronounced for long-term invasion success. Growth rate was the most important trait promoting invasion success, especially when the species invaded into a disturbed local community. We conclude that neither species traits nor environmental factors alone conclusively predict invasion success, but an integration of both of them is necessary.

## Introduction

Natural barriers such as mountains or oceans limit the dispersal of species. Due to increased mobility of humans [1], global trade [2] and climate change [3], however, various species get to regions outside their native range. Some of the alien species may be beneficial, like most intentionally introduced crop-plant species which serve as food for humans [4]. Many introduced species, however, exert strong negative impact on the new local environment by competition with or predation of native species. Wilcove et al. [5] concluded that 49% of all threatened or endangered species are on risk because of non-native species. Invasive species cause also a huge financial cost, adding up in the USA alone to more than $120 billion per year [1]. This just includes economic damages and control costs but not monetary values of ecological loss such as species extinctions or altered ecosystem services.

Given all these negative effects of invasive species, there is a strong incentive to understand the causes of successful invasions. It is commonly observed that of the many introduced species only few can establish, and even less become invasive [6]. Predicting the latter is a major goal in basic and applied ecology. Successful prediction of invasion success may eventually improve prevention, and lead to a better use of resources to mitigate the costs of invading species. Specifically, we would like to know why and which species become invasive, and others not. While there is a large and still increasing number of studies on invasive plants and animals, very little is known on invasion dynamics of microbes [7], [8], even though these organisms are often exhibiting key roles in natural ecosystems [9]. Only few studies looked at invasion dynamics of non-pathogenic microorganisms [10], and even fewer used a replicated experimental approach, which is necessary to identify general principles [8], [11].

For animals, plants and even microbes [7], there are generally two major perspectives used in explaining invasion processes: One line of thinking suggests that successful invaders have some trait characteristics that give them a deterministic advantage [7], [12], [13], [14]. In a recent meta-analysis of 117 studies comparing invasive and non-invasive confamilial plants, a clear difference between traits associated with high performance were observed in invasive and non-invasive plants [14]: the invasive species showed significantly higher growth rates, larger size or more leaf-area allocation than the non-invasive species. Other approaches went beyond species traits in the strict sense and looked at species interactions and the co-occurrence of locally adapted parasites and pathogens. For example, the enemy-release hypothesis suggests that the invaders have an advantage due to the loss of their co-evolved parasites in the new environment [15]. Torchin et al. [16] observed that introduced species have half the number of parasites compared to their native range and were also less heavily parasitized than native species.

A very different perspective in explaining invasion success is to focus on the invaded community, and not on the invading species only [7], [17]. It is generally recognized that certain communities are more at risk of being invaded than others. The most prominent example is geographically isolated islands that have lost many native species due to invasions. However, besides isolation also more subtle aspects of the local community or environment may promote or hinder invasions, such as environmental disturbances, native species richness or productivity [7], [18], [19]. Disturbance is often regarded as a key mechanism that permits an alien species to invade, as it reduces population density in the native community, potentially allowing the invaders to establish. There is empirical evidence that disturbance of the environment promotes invasion success [20], at least over short term time scales [21].

Given the immediate effects of biological invasions, the majority of studies on invasions are based on comparative field observations and case studies, mostly on plants and animals [16], [18], [22]. These studies are important as they provide direct information on real-world invasions and we can learn important principles from them. However, the benefit of realism comes with the cost of generally having only one realization of the process. Also, in nature we often just observe species that were able to persist while introduced species that fail to establish go mostly unnoticed. Only experiments allow a causal manipulation of factors, replication and the recording of failed invasions. However, intentional invasion experiments in the wild are unethical. Microcosm experiments with microorganisms are one way to go beyond the mentioned limitations [8], and also allow to generalize invasion principles beyond animals and plants [7]. Because microcosm experiments are done in the lab, failed establishment and failed invasions can be recorded. By using microorganisms it is possible to follow the invasion dynamics over tens of generations in an experiment lasting less than a month or making experiments that test a combination of species traits and community traits by manipulating invading species or the invaded community [8]. For example, Warren et al. [8] used microcosms with protists to address the predictability of invasion success of different species. More recently, the effect of productivity, invasion history and diversity of the invaded community on invasion success were looked at in microcosm experiments [11]. Such microcosm experiments can also address the stochastic component of invasion events [23]. With the exception of van Elsas et al. [11], to our knowledge, all of these studies have been using artificially assembled resident communities, and none of them was using clearly allopatric combinations of invaders and resident communities.

We conducted a microcosm experiment with aquatic protists and rotifers to causally understand factors influencing the success of biological invasions. We tested invasion success in response to a combination of traits of the invading species and environmental condition of the invaded community. We used nine invading species, originally isolated in North America, and followed replicated invasions into a natural European pond-derived community. We generally measured invasion success as the proportion of successful establishments based on presence/absence data across the replicated communities. The invading species differed in traits that are generally seen as important in explaining invasion success [7], [14], such as size, trophic level, or growth rate. We recorded both short-term and long-term invasion success. Furthermore, we either repeatedly disturbed the local invaded environment, or kept it as undisturbed control, to address the importance of environmental disturbances on invasion success.

Besides looking at invasion success, we also investigated subsequent effects on the local communities. Environmental disturbances as well as invasions may affect the composition and size distribution of the resident community. Changes in community composition or community size distribution, e.g., induced by disturbances, may also interact and affect the success of invasions. For example, an environmental disturbance may predominantly affect larger species [24]. This would shift the community size distribution and advantage larger species to invade, as their size-niche would have been freed. Furthermore, communities with a low diversity are more prone to invasions but invasions may also result in a loss of diversity in local communities [18], [19]. Thus, in many natural invasion-scenarios, the interest is not only to understand the invasion success but also predict the consequences for the local community. To better understand the effect of the invading species on the local community, we measured the size distribution of all organisms in the local communities, as well as community composition (Shannon diversity index) of a subset of local communities after successful invasions. These measures allowed us to quantify the effects of the invading species on the composition of the local communities.

## Materials and Methods

### Invading Species and the Natural Community

We used eight protist and one rotifer species as invading species. These were: *Blepharisma* sp., *Cephalodella* sp. (the rotifer), *Colpidium* sp., *Euglena gracilis*, *Euplotes aediculatus*, *Paramecium aurelia*, *Paramecium bursaria*, *Spirostomum* sp. and *Tetrahymena* sp. The rotifer and most of the protist species were originally collected from a natural pond in North America at Rutgers University [25], whereas *Blepharisma* sp., *Spirostomum* sp. and *Tetrahymena* sp. were supplied from Carolina Biological Supply Company (Burlington, North Carolina, USA), and were probably also originally isolated in North America. Even though some of these species have a cosmopolitan distribution, at least the genotypes, if not the species, used in the experiment are non-native to Europe. All invading species are obligate or facultative bacteriovore. *Blepharisma* sp., *Cephalodella* sp., *E. aediculatus* and *Spirostomum* sp. are also predators and able to feed on smaller protists. In the following, we call them “predator”, while the others are called “bacteriotroph”. Furthermore, *E. gracilis*, *E. aediculatus* and *P. aurelia* are able to photosynthesize. In the following text they are called “autotrophs” to distinguish them from invading species which are not able to photosynthesize and are therefore called “heterotroph”. For all species, we had previously measured species-specific traits, namely size, carrying capacity and growth rate [26].

**Figure 1:**
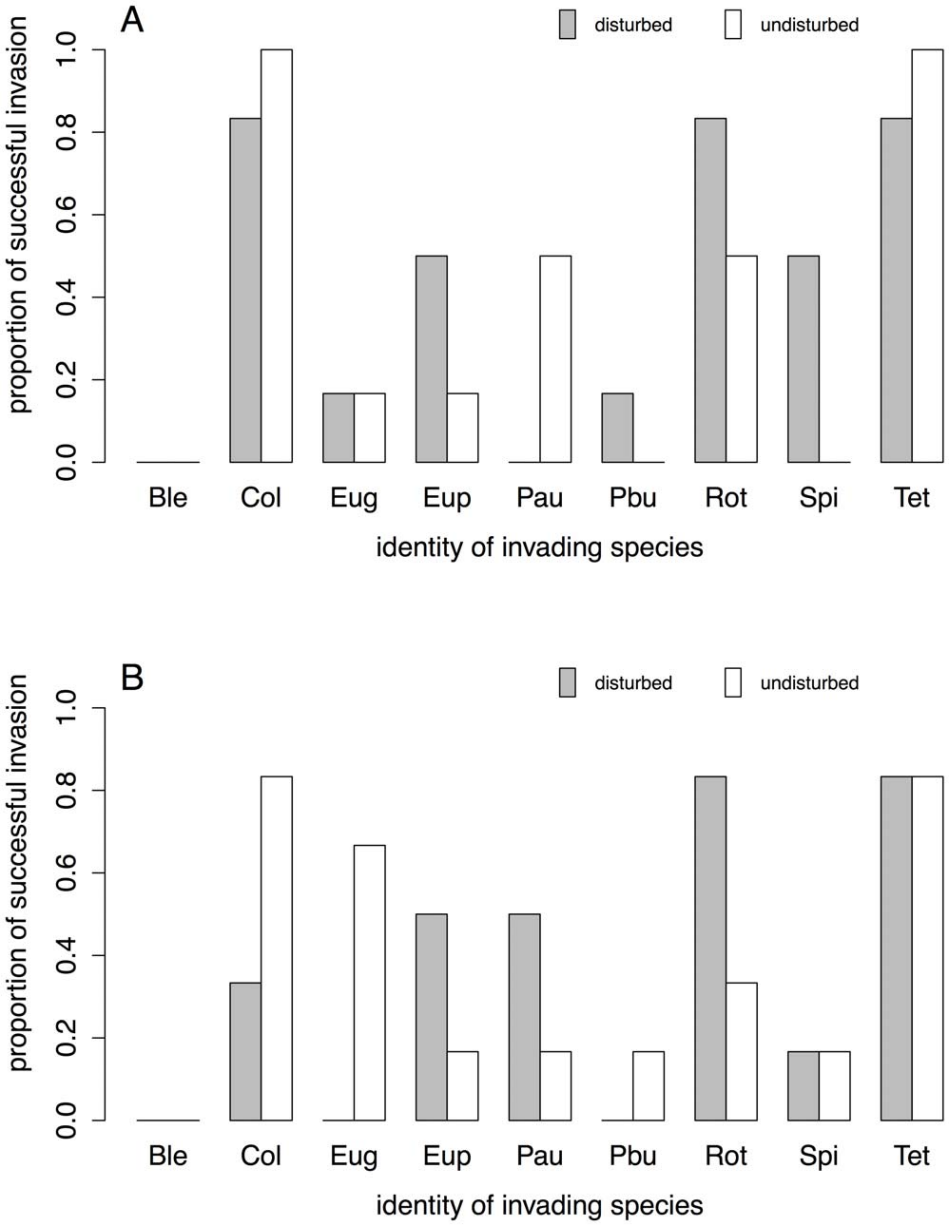
Invasion success of the nine invading protist and rotifer species in response to disturbance of the local environment. Invasion success is given as the proportion of communities in which the invading species was present, irrespective of the invading species’ density. A) initial and B) final measurement. Full species names of the abbreviations are given in the Material and Method section.

**Table 1:** GLMs on the effect of species identity of the invader, disturbance of the local community and their interaction on invasion success.

	effect	df	deviance	resid. df	resid. deviance	F	P
a) Initial measurement	Invader	8	56.2	99	89.0	9.6	<0.0001
	Disturbance	1	0.6	98	88.4	0.9	0.355
	Interaction	8	17.3	90	71.1	2.9	0.006
	Null			107.0	145.2		
B) Final measurement	Invader	8	34.3	99	106.9	4.6	<0.0001
	Disturbance	1	0.1	98	106.9	0.1	0.808
	Interaction	8	18.7	90	88.2	2.5	0.017
	Null			107.0	141.3		

Invasion success was used as a binary response variable in the models. It described if the invading species was present or not in a community, irrespective of the invading species’ density. GLMs were done with a quasibinomial error distribution, and subsequent F-significance testing.

We grew individual pure cultures of the invading species in a standardized protist medium. The medium consisted of 0.46 g Protozoan Pellet (Carolina Biological Supply Company) in 1 litre of tap water. It was autoclaved and cooled down before use. Previous to the main experiment, we grew all invading species in pure cultures. Each of these cultures was kept in a glass jar with 100 ml of medium. Two wheat grains were added to the jars to serve as carbon source for bacteria, which on their part figured as food source for the bacteriovore protist community. We covered the cultures with aluminium foil. These cultures served as source populations of the invading species. A characteristic of invasions is that the invading species is entering a new environment outside its natural range. Thus, the invading species is not only encountering another community, but also potentially different environmental conditions compared to its native environment. Accordingly, we collected a resident community of protists, rotifers and microbes together with the water from a natural pond. The pond where we collected the natural community is situated in Pfäffikon ZH, Switzerland (location: 47° 22′ 28.32″ North, 8° 48″ 08.35″ East). We confirmed that all invading species used in our experiment were morphologically distinguishable from the protist and rotifer community in the natural pool. We filtered the pond water through a filter with mesh size of 250 µm, to exclude bigger aquatic invertebrates. Then we filled each of 120 jars with 100 ml of the pool water and the natural microbial community and placed them into an illuminated climate chamber at a temperature of 20°C. After one week we added a single invading species to each jar. For every invading species we had 6 replicates of disturbed and 6 replicates of undisturbed patches (see paragraph below on disturbances). Invasions started with an initial population size of about hundred individuals per invading species and patch, except for *Spirostomum* sp., which occurs generally at much lower densities. In this case we added 25 individuals per patch. The invading species were added within 2 ml of medium. As a control, we had replicates that were containing the natural microbial community, and where we added 2 ml of autoclaved protist medium without an invading species. In a few jars we found *Chironomidae* larvae after the onset of the experiment. They probably hatched from eggs that passed the filter, and we consequently removed them.

**Figure 2:**
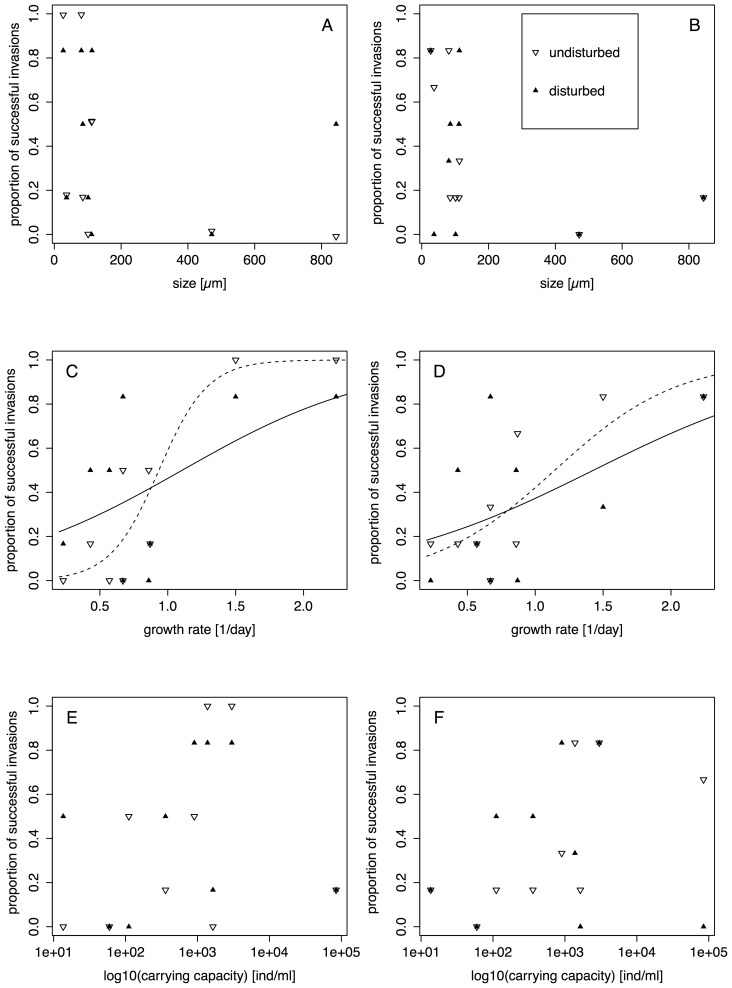
Correlation between invasion success and traits of the invading species. Traits concern size (A, B), growth rate (C, D) and carrying capacity (E, F). Left hand panels are the results of the initial measurement and right hand panels are the results of the final measurement. Invasion success is given as the proportion of communities in which the invading species was present, irrespective of the invading species’ density. For significant correlations, we added lines of the values predicted by the GLM. Dashed lines are for undisturbed patches, solid lines for disturbed patches.

**Table 2:** GLMs on the effect of species traits of the invading species and disturbance of the local community on invasion success.

	effect	df	deviance	resid. df	resid. deviance	F	P
A) Size, initial measurement	Size	1	8.0	16	66.1	2.18	0.161
	Disturbance	1	0.4	15	65.7	0.10	0.754
	Null			17	74.1		
B) Size, final measurement	Size	1	10.4	16	42.6	4.38	0.054
	Disturbance	1	0.0	15	42.6	0.02	0.894
	Null			17	53.1		
C) Growth rate, initial measurement	Growth rate	1	32.6	16	41.5	14.40	0.002
	Disturbance	1	0.5	15	41.1	0.21	0.651
	Null			17	74.1		
D) Growth rate, final measurement	Growth rate	1	19.5	16	33.5	9.84	0.007
	Disturbance	1	0.0	15	33.5	0.02	0.877
	Null			17	53.1		
E) Carrying capacity, initial measurement	Carrying capacity	1	2.7	16	71.3	0.75	0.401
	Disturbance	1	0.4	15	71.0	0.10	0.760
	Null			17	74.1		
F) Carrying capacity, final measurement	Carrying capacity	1	0.0	16	53.1	0.01	0.962
	Disturbance	1	0.0	15	53.0	0.01	0.910
	Null			17	53.1		

Proportion of invasion success was used as the response variables (odds ratio). GLMs were done with quasibinomial error distribution, and subsequent F-significance testing. Models were separately conducted for all traits, and both the initial/final measurement (A–F).

**Figure 3:**
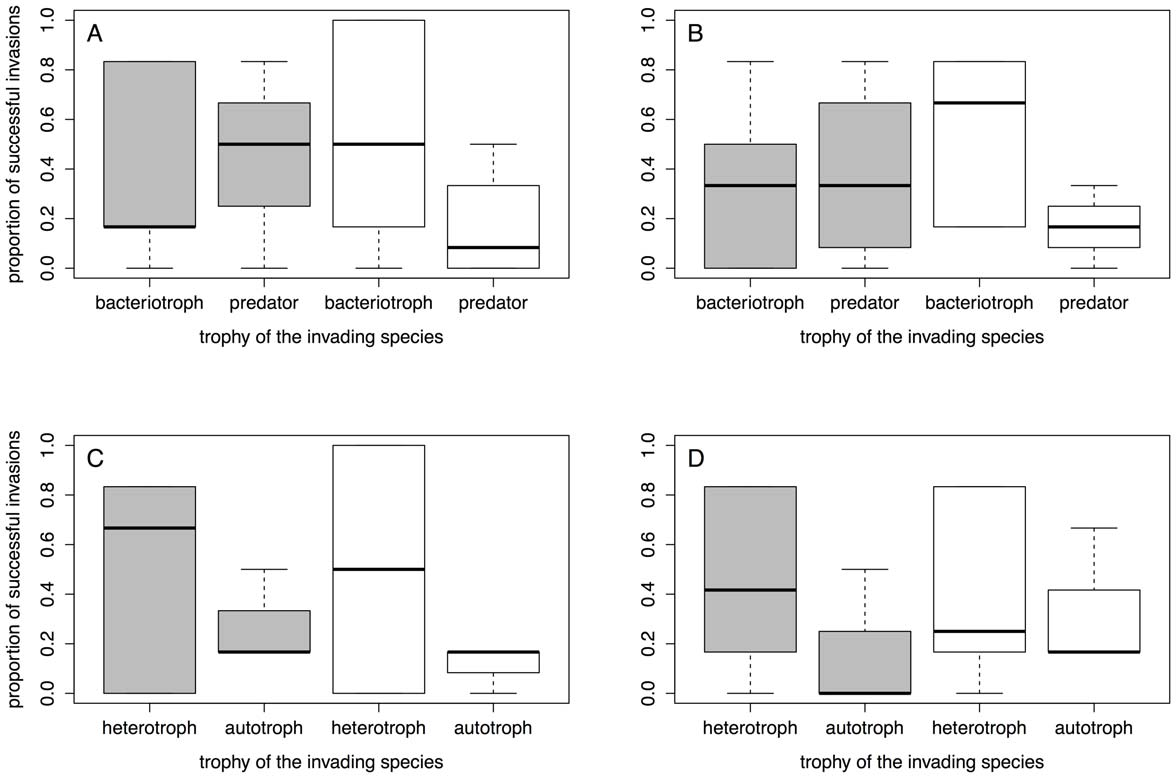
Invasion success in response to the invading species’ trophy. Boxplots are given separately for predators vs. bacteriotrophs (A, B) and autotrophs vs. heterotrophs (C, D). Invasion success is given as the proportion of communities in which the invading species was present, irrespective of the invading species’ density. Grey boxes stand for disturbed patches, white boxes stand for undisturbed patches. Left hand panels show results of the initial measurement, and right hand panels show results of the final measurement.

**Table 3:** GLMs on the effect of trophy and disturbance of the local environment on invasion success.

	effect	df	deviance	resid. df	resid. deviance	F	P
A) Predator, initial measurement	Predator	1	2.7	16	71.4	0.71	0.412
	Disturbance	1	0.4	15	71.1	0.10	0.762
	Null			17	74.1		
B) Predator, final measurement	Predator	1	3.1	16	50.0	1.08	0.315
	Disturbance	1	0.0	15	49.9	0.01	0.906
	Null			17	53.1		
C) Autotroph, initial measurement	Autotroph	1	9.9	16	64.2	3.05	0.101
	Disturbance	1	0.4	15	63.8	0.12	0.737
	Null			17	74.1		
D) Autotroph, final measurement	Autotroph	1	3.0	16	50.1	1.05	0.323
	Disturbance	1	0.0	15	50.0	0.01	0.906
	Null			17	53.1		

Proportion of invasion success was used as the response variables (odds ratio). GLMs were done with quasibinomial error distribution, and subsequent F-significance testing. Models were separately conducted for all traits, and both the initial/final measurement (A–D). A and B compares predatory species with bacterotrophic species, and C and D compares autotrophic with heterotrophic species.

During all of our work, we took precautions to prevent the spread of protists into natural environments and the experiment was conducted in accordance with all legal regulations concerning non-native species and laboratory security.

**Figure 4:**
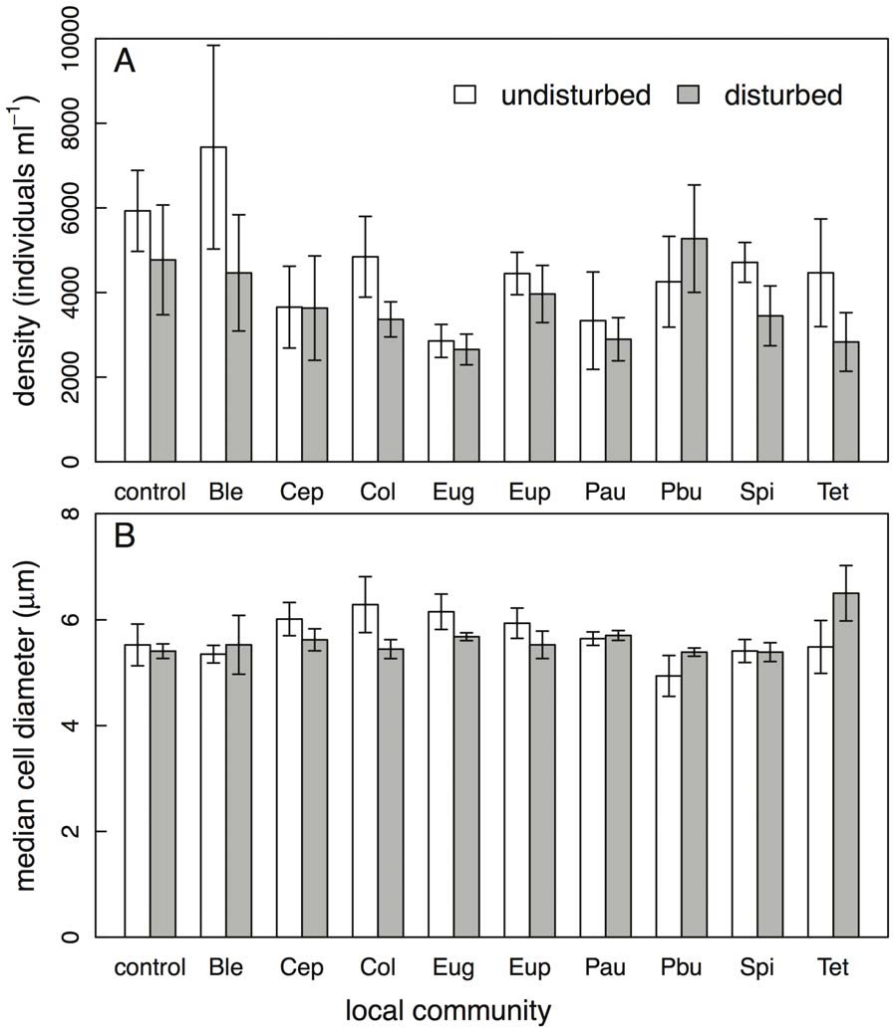
Density and median size of all microorganisms (protists and rotifers) in the individual communities. Density (A) and size distribution (B) was measured with a CASY particle counter, and includes all individuals between 3.2 µm and 120 µm. Mean±SE values are given separately for replicates with different invading species and disturbance levels. For full species names see Material and Method section.

**Figure 5:**
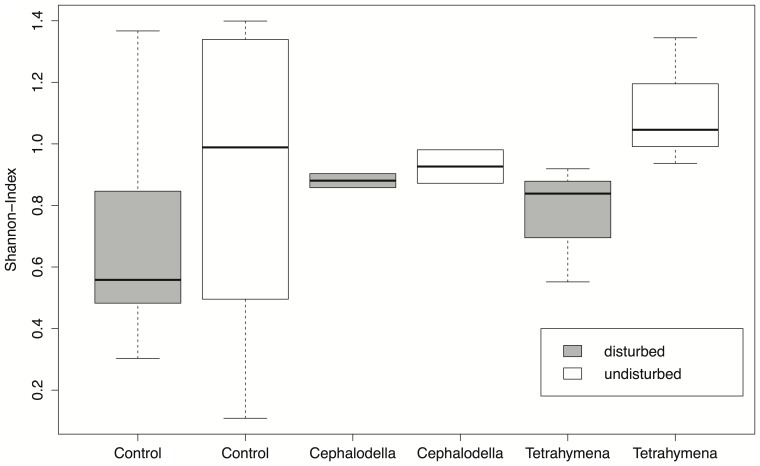
Community diversity (Shannon-index) in response to disturbance of the local environment and the invading species. Boxplots are given for the control communities without invasions, and the communities invaded either by *Cephalodella sp.* or *Tetrahymena sp.*

### Disturbance Events

We were interested in the invasion success as a response of species traits of the invading species and the occurrence of environmental disturbances in the invaded patch. Therefore, we created for every invading species disturbed and undisturbed patches. To simulate disturbance events we repeatedly applied 95% density reductions. We had in total four disturbance events occurring on a weekly interval. This disturbance level has already been used successfully in other experiments [27], [28]. Disturbance-induced mortality of the microorganisms was achieved by putting 95% of the well-mixed culture into a microwave for 5 minutes at 800 W. Afterwards the medium cooled down to room temperature and was given back to the patch within 45 minutes. Thereby, we kept the composition of the pond water constant and avoided nutrient addition or loss. We adjusted evaporation of the pond water with deionised water. It is not only hypothesized that invasion success may be higher in disturbed habitats, but also that many invasions are intrinsically linked to the disturbances, such that the same factor is disturbing the environment and bringing in a non-native species. For example, Lozon and Maclsaac [29] found that the establishment of exotic species is associated with disturbances in 56% of the invasion events (68% of the plant invasions, and 28% of the animal invasions). Hence, we added the invading species immediately after the first disturbance.

### Measuring Invasion Success

We measured invasion success at two time points, the first reflecting short-term establishment success, the latter long-term persistence. Short-term success was measured five days after the addition of the invading species (i.e., a little less than two weeks after the onset of the experiment, shortly before the second disturbance occurred). Long-term success was measured five days after the last disturbance (i.e., five weeks after the onset of the experiment). Our main response variable was presence/absence of the invading species. When not stated differently, the term “invasion success” describes the proportion of communities in which the invading species was present, irrespective of its density. We measured the population densities of the invading and resident species, to get additional information on the effect of the invading species on the resident community.

We screened a defined subsample of each replicate under a stereo-microscope to estimate the presence/absence and density of the invading species. For the measurement of short-term success, we screened a maximum volume of 1 ml. For the final measurement we screened different volumes for each invading species; the volume screened was optimised for each species, to avoid false-negative measurements, for details see [27], [30]. For *Spirostomum* sp. we screened 10 ml, for *Blepharisma* sp. and *P. bursaria* 5 ml, for *E. aediculatus* and *P. aurelia* 3 ml, for *Colpidium* sp. 2 ml and for *Cephalodella* sp., *E. gracilis* and *Tetrahymena* sp. 1 ml. The jars were mixed properly before taking subsamples. We calculated the density of the invading species per ml within each replicate.

To get an overview of the local community structure we took further measurements at the end of the experiment (five weeks after the onset of the experiment; i.e., five days after the last disturbance and measured at the same time as the long-term invasion success) and analysed diversity of the local community in patches where the invading species could persist in the long term. First, we screened the control communities containing only the natural community. In those samples we classified as many morphospecies as possible. We then focused on 10 of these categories of different species and species groups that could be distinguished easily with a stereo-microscope. As previously described, we recorded their density by screening a maximum volume of 2 ml. The relative abundance of these species was used to calculate a diversity measure for each community (Shannon-index). For some categories we were only able to get rough estimations because of their high densities. Due to time-constraints, we limited this analysis to the controls without invasions, three disturbed and three undisturbed patches with successful *Tetrahymena* sp. invasions, and two disturbed and two undisturbed patches with successful *Cephalodella* sp. invasions.

Finally, we measured the size distribution of the whole microbial community for each replicate with a particle counter (CASY-counter, Model TTC, Roche Diagnostics AG, Switzerland; 150 µm capillary) at the final measurement. This measurement gives a standardized and highly resolved size distribution of all particles with a volumetric size equivalent to spheres with a diameter ranging between 3.2 µm and 120 µm.

### Statistical Analysis

We analysed the invasion success (successful establishment based on presence absence data) of the individual species with generalized linear models (GLM), using disturbance and species identity as predictor variables. The binary response variable described invasion success or failure in each of the 108 replicated communities (excluding the controls) at the initial and the final measurement. For the GLM we used a quasibinomial link function, as we had some overdispersion in the model, and a F-significance test, following the approach recommended by Crawley [31]. Furthermore, we used GLMs to test the effect of species traits (size, carrying capacity, growth rate and trophic level), environmental disturbance and their interaction on invasion success, described as odds ratio of successful invasions (i.e., again based on presence-absence data). To analyse our proportion data, we used a quasibinomial link function, and an F-significance test, as recommended for model simplification and significance testing of individual factors [31]. Residual deviances of models were used as the goodness-of fit criterion in the evaluation of the models. We compared the density of the invading species with their carrying capacity measured in isolation. Density varied a lot not only between different species but also within species and across sampling events (temporal variation, data not shown), and we therefore did not use it as a measure of invasion success.

We calculated the Shannon-index for the patches where we studied the diversity of the local community, now using density data and not only presence/absence data. Again, we compared disturbed patches with undisturbed patches for each different treatment, using an ANOVA. We also analysed the size distribution data (CASY-measurements) with ANOVAs, using total density and mean size of all protist and rotifer species in the communities as response variables, and species identity of the invader and environmental disturbance as explanatory variable. We assured that the normality assumptions of ANOVA/ANCOVA models were fulfilled in all analyses with these continuous response variables. Model simplification and significance testing were done accordingly to Crawley [31]. All statistical analyses were done with the program R version 2.12.1 (R Development Core Team 2008).

## Results

Invasion and establishment success, based on presence/absence data, of the individual species was significantly affected by the identity of the invading species, both at the initial and final measurement (Fig. 1, Table 1). Disturbance as a main factor was not significantly promoting invasions. However, we found a temporally consistent and highly significant interaction between the invading species’ identity and disturbance (Fig. 1), meaning that for some species environmental disturbances increased invasion success, while decreasing it for others.

There was a significant positive correlation between invasion success (proportion of invaded communities) and growth rate of the invading species, both for the initial (Fig. 2C, Table 2C) and the final measurement (Fig. 2D, Table 2D). All other species traits did not significantly correlate with invasion success (Table 2). None of the interactions between the species traits and disturbance was significant. We also found no significant difference in the invasion success of invading predator versus invading bacteriotroph species. The same was true for the comparison of invading autotroph versus invading heterotroph species. Additionally, there was no significant interaction between the trophic levels and disturbance (Fig. 3, Table 3A,B). Density of the invading protists varied over more than four orders of magnitude, and carrying capacity measured in isolation [26] was not a good predictor of the densities observed in the communities. There were no significant correlations between carrying capacity measured in isolation and mean density after successful invasion at the initial or final measurement or maximal observed density of a species (rank correlation test, all *P*>0.2).

The density of microorganisms in the invaded communities was significantly affected by the identity of the invading species (*F*_8,90_ = 2.1, *P*  = 0.05, Fig. 4). Disturbed communities had a marginally significant lower density of organisms (*F*_1,90_ = 3.4, *P*  = 0.07), while there was no significant interaction among these two factors on density (*F*_8,90_ = 0.8, *P*  = 0.6). Mean size of protist and rotifer species in the different communities was neither affected by the invading species’ identity (*F*_8,90_ = 1.7, *P*  = 0.12, Fig. 5), nor the occurrence of environmental disturbances (*F*_1,90_ = 0.03, *P*  = 0.86), and also the interaction was non-significant (*F*_8,90_ = 1.6, *P*  = 0.14).

Finally, there was no significant difference in the Shannon-index describing community composition in disturbed versus undisturbed patches (*F*_1,18_ = 1.94, *P*  = 0.18, Fig. 5). There was also no significant difference in the Shannon-index for different invading species (*F*_2,18_ = 0.43, *P*  = 0.66, Fig. 5).

## Discussion

Our invasion experiment demonstrated that invasion dynamics in microorganisms (Fig. 1) may be driven by similar factors as in animal and plant species, namely by environmental disturbances and species traits of the invading organisms [13], [14], [20], [21], [29]. The significance of these two factors [20] on microorganisms’ invasion dynamics has been predicted [7], but never been tested in an experiment that used realistic combinations of resident communities and invading species. Interestingly, we found that disturbances did not have consistent effects for all species, and, depending on the invading species identity, a disturbance either promoted or decreased invasion success (Fig. 1, Table 1). The interaction between disturbances and species’ identity explained initial establishment success, but was especially pronounced for long-term success [7]. The observed interaction may be generally valid for other taxonomic groups, and not restricted to microorganisms, as our experiment was not confining biological aspects to a specific group of organisms. Importantly, with our study we were not only replicating the invasion events, but also avoided that the disturbance treatment was confounded with a manipulation of other environmental factors, such as a change in overall availability of nutrients or resources [21]. We see a disturbance as a sudden change in environmental conditions that opens up niche space [24]. Nutrients in the system may become available, but otherwise the system is closed and the overall nutrient level stays constant. We intentionally kept nutrient levels constant at both control and disturbance treatment and thereby avoided that a nutrient loss or gain would be intrinsically linked with disturbances. Our results suggest that the significance of species identity in explaining invasion success is probably driven by a different exploitation of the local resources by different species compared to the resident species, and not due to a preference of other (i.e., higher) nutrient levels only.

Previous experiments and comparative studies on invasion processes focussed mostly on animal and plant species [13], [14], [16], [32], or were studying the invasion into artificially composed resident communities, often in grasslands [14], [21]. This bias is mostly due to our limited knowledge on real invasions of microorganisms [7], especially in aquatic or soil systems. While hard to study, the consequences of such invasions can be devastating. For example, the crayfish *Procambarus clarkii* is a classic example of a well-studied, large animal species invading many areas. It has its origin in North America and invaded Europe within the 20^th^ century, where it is threatening native crayfish species due to its competitive ability, but even more so due to a microbial fungal disease that was brought along with the crayfish [33]. Thus, the microorganisms (i.e., the fungus) is probably having as high or even higher consequences as the “large” crayfish itself. When the crayfish was intentionally introduced into Europe, the living animals obviously came along with water from their natural habit. It is thus very likely that it not only came along with a fungal disease [33], but also with many other microorganisms such as protists, rotifers or aquatic nematodes that were attached to the crayfishes’ carapace or in the water transported along with it. This is exemplifying a possible invasion scenario for microorganisms, which may be rather common but rarely studied [7]. Studies on invasion dynamics of microorganisms, including ours, not only address the dynamics of such invasions of microorganisms, but also take advantage of the small scale of microbial systems to conduct experiments [8], [11]. Compared to previous studies, we used a realistic scenario of native and invading species (native resident community collected in Europe, invading species originally collected in North America), and the invasion scenario is thereby in close geographical accordance to many invasions happening in nature [32]. While species identity (Fig. 1) and species traits (Fig. 2) explained invasion success overall, we still observed a high variability among the intraspecific replicates. Only one species (*Blepharisma*) had a consistent (negative) invasion success, and failed to establish in all cases. All other species varied in their invasion success between 20 to 80%, and none of the species invaded 100% of the resident communities. Also other studies, such as Warren et al. [8], found in similar microcosm experiments (using, however, artificially composed resident communities) that long term success was not only different among species, but also not uniform within the intraspecific replicates. This suggests a strong intrinsic, stochastic component on invasion success [23].

While invasion success into individual replicates was not deterministic with respect to species identity, we found, in accordance with other studies [8], for many species a consistent outcome between short term establishment success and long-term persistence. Of the 108 invasion events, 55 resulted in a short-term establishment, and of those 39 persisted over the long term. The consistent success in both short and long-term perspective was for example found for *Cephalodella* sp., *Colpidium* sp. (just undisturbed patches), *Euplotes* sp. and *Tetrahymena* sp. Such a high persistence after initial establishment also suggests that the most critical phase during an invasion process in microbial communities is during early establishment, while once established, the invaded species face fewer challenges by the local community. This of course has important conservation and management implications, as it highlights the significance of early counter-measures against non-native species.

For a better understanding of the various outcomes among species, we looked at species’ traits. Our results suggest that growth rate is the most important species trait affecting invasion success into a disturbed community (Fig. 2). Haddad et al. [34] have shown that among protist species, those with a high growth rate can persist better in disturbed communities in a microcosm experiment. We extend this finding and showed that it is not only relevant in “artificially” composed communities, but also for invaders into a natural resident microbial community of protists and rotifers. While the link between a high growth rate and a fast recovery after disturbance events may seem evident [14], it is not universal and Thompson et al. [21] concluded from a long term experiment that no single trait, including growth rate, acts as a good predictor for invasiveness of a species. This, as many other studies, however, was a study conducted on plants, and the relationship between traits and invasion was followed over a relatively short time of at maximum five generations of the invading organisms [13], [14], [21]. In our experiment we covered at least 20 to 40 generations, and applied multiple sequential disturbance events. As such, our experimental result coincides with a comparative study on invasive cyanobacteria and protists [35], which found that the invasive species have a higher growth rate compared to native microorganisms occupying a similar niche.

Surprisingly, we did not find any correlation between further species’ traits and invasion success. Some [14], but not all [13], studies on plants suggest that invasive species tend to be bigger than local species, while we did not find an effect of the microorganisms’ size on invasion success. The size effect in plants could be due to a bias of larger plant species being more often introduced for horticulture because of their big flowers, such that size as an explanatory factor is an artefact and not a causality [22]. By using a random set of species as invaders, we could avoid such a bias. However, size as specific trait could have also been confounded with other traits, such a trophic level of the protists, thus making a comparison more difficult. We could also not find a correlation between carrying capacity of the invading species measured in isolation and their invasion success. Species reaching higher population numbers may be less affected by environmental stochasticity, and thus have a better chance of persistence. However, many of the invading species did not reach their carrying capacity (as measured in isolation) during the experiment. Consequently, the response variable may not be fully reflecting their actual population dynamics. Finally, trophic level did not predict invasion success. We expected that there would be an advantage for autotrophic species, especially in disturbed patches, as they are not depending on prey species recovering too from the disturbances. However, the three autotrophic species may not have had optimal light conditions in the experiment and so the potential advantage of the autotrophic species could have been reduced. The generally low predictability of invasion success using species traits of our invading species may also be that these traits were measured in a standardised protist medium, and not in the environment in which the species invaded. Hence, it could be that trait expression was different in the experiment, which would reduce predictive ability. This can be a general issue in studies on invasion success, where it is observed that trait values of a species may be different when measured in the native versus the non-native range.

At the end of the experiment, we measured how disturbances and invasions were affecting the local community. Overall, undisturbed communities had a significantly higher density of microorganisms compared to disturbed patches (Fig. 4), and there was also a tendency of a higher diversity in undisturbed patches (Fig. 5). However, these effects were not very strong and we could not find a difference in the diversity of the local community when the invading species were successfully persisting and the no-invasion controls (Fig. 5). This indicates that the invading species integrated into the local communities without causing large shifts in the resident’s community structure. This is also supported by the local community size distribution, which neither changed in response to disturbance nor invasion of different species. We thus conclude that our natural microbial community seemed not very vulnerable to be strongly negatively affected by new protists and rotifers arriving, even for naturalistic invasion scenarios. In accordance, Warren et al. [8] could not observe an association between a species’ likelihood of establishment and its potential to alter the resident community. It suggests that our natural community of protists and rotifers was not saturated with species, and empty niches were available for invading species.

We conclude that environmental disturbances, even if often highlighted, are not exclusively promoting invasion events. Rather, we need to look at interactions of environmental disturbances with species identity. Our experiment identifies intrinsic growth rate (measured in isolation) as the most important specific trait defining invasion success. While this has also been observed in other studies, further experiments are needed to get a deeper understanding of this relationship, and especially if a trade-off between growth rate and competitive ability might interfere with these predictions. Microcosm experiments such as ours can be an excellent tool for disentangling the individual components. Ideally, the outcome of microcosm experiments can then be compared with results from comparative and possibly experimental field studies, on both macro- and micro-organism, to get a better understanding of the complexity of invasion processes.
